# Characterization and Plasmid Elimination of NDM-1-Producing *Acinetobacter calcoaceticus* from China

**DOI:** 10.1371/journal.pone.0106555

**Published:** 2014-09-02

**Authors:** Yang Sun, Qi Liu, Shuo Chen, Yang Song, Jun Liu, Xuejun Guo, Lingwei Zhu, Xue Ji, Lizhi Xu, Wei Zhou, Jun Qian, Shuzhang Feng

**Affiliations:** 1 Institute of Military Veterinary, AMMS, Key Laboratory of Jilin Province for Zoonosis Prevention and Control, Changchun, China; 2 Beijing Institute of Genomics, Chinese Academy of Sciences, Beijing, China; Cornell University, United States of America

## Abstract

The presence of multidrug-resistant bacterial pathogens in the environment poses a serious threat to public health. The opportunistic *Acinetobacter* spp. are among the most prevalent causes of nosocomial infections. Here, we performed complete genome sequencing of the *Acinetobacter calcoaceticus* strain XM1570, which was originally cultivated from the sputum of a patient diagnosed with pneumonia in Xiamen in 2010. We identified carbapenem resistance associated gene *bla*_NDM-1_ located on a 47.3-kb plasmid. Three methods – natural reproduction, sodium dodecyl sulfate treatment and nalidixic acid treatment – were used to eliminate the *bla*_NDM-1_-encoding plasmid, which achieved elimination rates of 3.32% (10/301), 83.78% (278/332), and 84.17% (298/354), respectively. Plasmid elimination dramatically increased antibiotic sensitivity, reducing the minimum bacteriostatic concentration of meropenem from 256 µg/ml in the clinical strain to 0.125 µg/ml in the plasmid-eliminated strain. Conjugation transfer assays showed that the *bla*_NDM-1_-containing plasmid could be transferred into *Escherichia coli* DH5α:pBR322 *in vitro* as well as *in vivo* in mice. The *bla*_NDM-1_ genetic environment was in accordance with that of other *bla*_NDM-1_ genes identified from India, Japan, and Hong-Kong. The multilocus sequence type of the isolate was identified as ST-70. Two novel genes encoding intrinsic OXA and ADC were identified and named as OXA-417 and ADC-72. The finding of *bla*_NDM-1_ in species like *A. calcoaceticus* demonstrates the wide spread of this gene in gram-negative bacteria which is possible by conjugative plasmid transfer. The results of this study may help in the development of a treatment strategy for controlling NDM-1 bacterial infection and transmission.

## Introduction

The widespread clinical use of antibiotics has induced rapid evolution of the amount and types of multidrug-resistant bacteria. Antibiotic resistance, which is acquired mainly by conjugative plasmid DNA transfer, is a threat to global health. In 2009, New Delhi metallo-β-lactamase (NDM-1), which confers high carbapenemase activity, was first detected in clinical samples from a Swedish patient of Indian origin in New Delhi, India [1]. Bacteria carrying the NDM-1-encoding *bla*_NDM-1_ gene are resistance to a broad spectrum of β-lactam antibiotics, including carbapenems, which are frequently used to treat infections caused by antibiotic-resistant gram-negative bacteria [2]–[4]. Most *bla*_NDM-1_ genes are located in plasmids [5] and bacterial conjugation is a key lateral DNA transfer mechanism, allowing easy transmission between different bacterial pathogens. The Swedish patient was found to be simultaneously infected with *Klebsiella pneumoniae* and *Escherichia coli*, both of which carried a plasmid containing the *bla*_NDM-1_ gene [1].

*Acinetobacter* spp. are nosocomial pathogens that have become increasingly common worldwide over the past few decades and account for the majority of human infections and hospital outbreaks [6]. Relevant species have been grouped together as the *Acinetobacter calcoaceticus*–*Acinetobacter baumannii* complex (*A. baumannii*, *A. calcoaceticus*, *A.pittii* and *A*. *nosocomialis*). However, it is difficult to identify these species clearly by routine diagnostic methods [7]. Clinical isolates of the *A. calcoaceticus–A. baumannii* complex are frequently resistant to last-line antibiotics, including cephalosporins, aminoglycosides, fluoroquinolones, and carbapenems [8], [9].

The lack of efficient drugs against multidrug-resistant pathogens poses a serious threat to public health. One feasible method to address this threat is to eliminate the drug-resistance plasmid, as such plasmids may be occasionally lost during natural reproduction or may be eliminated by exposure to physical or chemical treatments [10]–[12]. Here, we characterized a NDM-1-producing *A. calcoaceticus* isolate from China using whole genome sequencing and plasmid elimination assays.

## Results

### Basic Identification and Antimicrobial Susceptibility Testing

The multidrug-resistant pathogen XM1570 was isolated from a sputum culture and analyzed using the Vitek 32 microbial identification/susceptibility system (bioMérieux Inc., Durnam, NC, USA) with the Panel GNI+, which identified the isolate as a member of the *A. calcoaceticus–A. baumannii* complex. Mass spectrometry showed that strain XM1570 could be *Acinetobacter* sp Genospecies 3 (score value 2.322), *A. calcoaceticus* (score value 1.968), or *A. baumannii* (score value 1.96). The *Acinetobacter* strain XM1570 was resistant to all tested β-lactam antibiotics, including imipenem, meropenem, ertapenem, cefazolin, ceftazidime, cefotaxime, cefepime, aztreonam, ampicillin, and piperacillin. It was also resistant to chloramphenicol, but remained susceptible to amikacin, ciprofloxacin, tetracycline, trimethoprim–sulfamethoxazole, and colistin (Table 1).

**Table 1:** Susceptibility results of the *A. Calcoaceticus* XM1570, transconjugant (the *E. coli* DH5α:pBR322 strain after successful transformation), *E. coli* DH5α:pBR322, and plasmid-cured strain.

Antimicrobial agent	MIC (μg/ml)			
	XM1570	transconjugant	DH5α:pBR322	plasmid-cured strain
Imipenem	>8 (R)	>8 (R)	< = 1 (S)	< = 1 (S)
Meropenem	>8 (R)	>8 (R)	< = 1 (S)	8 (I)
Cefazolin	>16 (R)	>16 (R)	>16 (R)	>16
Ceftazidime	>16 (R)	>16 (R)	< = 1 (S)	16 (I)
Cefotaxime	>32 (R)	>32 (R)	< = 1 (S)	32 (I)
Cefepime	>16 (R)	>16 (R)	< = 2 (S)	8 (S)
Aztreonam	>16 (R)	< = 2 (S)	< = 2 (S)	>16(R)
Ampicillin	>16 (R)	>16 (R)	>16 (R)	>16
Piperacillin	>64 (R)	>64 (R)	>64 (R)	32 (I)
Amoxicillin-Clavulanate	>16/8 (R)	>16/8 (R)	>16/8 (R)	>16/8(R)
Ampicillin-Sulbactam	>16/8 (R)	>16/8 (R)	>16/8 (R)	8/4 (S)
Piperacillin/Tazobactam	>64/4 (R)	>64/4 (R)	>64/4 (R)	32/4 (I)
Colistin	< = 0.5 (S)	< = 0.5 (S)	< = 0.5 (S)	< = 0.5 (S)
Trimethoprim-Sulfamethoxazole	< = 0.5/9.5 (S)	< = 0.5/9.5 (S)	< = 0.5/9.5 (S)	1/19 (S)
Chloramphenicol	>16 (R)	< = 4 (S)	< = 4 (S)	>16
Ciprofloxacin	< = 0.5 (R)	< = 0.5 (S)	< = 0.5 (S)	< = 0.5 (S)
Levofloxacin	< = 1 (S)	< = 1 (S)	< = 1 (S)	< = 1 (S)
Moxiflocacin	< = 1 (S)	< = 1 (S)	< = 1 (S)	< = 1
Tetracycline	< = 2 (S)	>8 (R)	>8 (R)	< = 2 (S)
Amikacin	< = 8 (S)	< = 8 (S)	< = 8 (S)	< = 8 (S)
Gentamicin	< = 2 (S)	< = 2 (S)	< = 2 (S)	< = 2 (S)
^Δ^imipenem	256	128	0.25	0.0625
^Δ^meropenem	256	256	0.0625	0.125
^Δ^ertapenem	>512	128	0.03125	2

Note: MIC  =  minimum inhibitory concentration, R  =  resistant, S =  susceptible, I =  intermediate. MICs are based on the BD automated microbiology system (Panel NMIC/ID-4). ^Δ^MICs are based on the broth microdilution susceptibility method following the standards of the Clinical Laboratory Standards Institute (document no. M100-S22, 2012).

### Molecular and Genomic Analyses

PCR screening and sequencing revealed the presence of *bla*_NDM-1_ in strain XM1570. The genome of strain XM1570 was completely sequenced (Genbank accession number: AMXH01000000). We obtained 87 contigs with a total length of 4,076,308 bp, including one 47.3-kb circular contig identified as a *bla*_NDM-1_- containing plasmid (AMXH01000087.1) and another 93.8-kb circular plasmid (CM001803.1). A total of 3,959 coding sequences were identified on the contigs. The *bla*_NDM-1_ region in the plasmid was located in an island of medium G+C content (40.83%); the average G+C content of the strain was 38.82%.

Sequence analysis of the *gyrB* gene showed that strain XM1570 had the highest homology with *A. calcoaceticus* PHEA-2 (98%). Analysis of *ropB* showed that the strain had the highest homology with *Acinetobacter* genomosp. 3 strain CIP 70.15 (99%) and *A. calcoaceticus* PHEA-2 (98%). The complete genome of strain XM1570 showed ∼96% nucleotide homology to *A. calcoaceticus* PHEA-2 (CP002177.1). Taken together, these results indicated that XM1570 should be designated as species *A. calcoaceticus*.

The gene encoding the intrinsic OXA enzyme was identified in the whole genome sequence, with inclusion of a fragment of insertion sequence *ISAba22* after the first 470 nucleotides of the *bla*_OXA_ gene. The length of the inserting sequence was 1277 bp. The OXA-type was assigned OXA-417 (http://www.lahey.org/studies/; KM220587), which represented the *bla*_OXA-213-like_ gene of *A. calcoaceticus* and shared 89.1% identity (30 amino acid substitutions) with *bla*_OXA-213_ protein (JN861781.1). Besides OXA and NDM-1, we identified a novel ADC gene in the genome (chromosome), which encoded an enzyme with a moderate degree of variability that was named *bla*_ADC-72_ (KJ885607). Sequence analysis of the *bla*_ADC-72_ gene showed that strain XM1570 shared 95% homology with *A. calcoaceticus* PHEA-2. The closest match was ADC-42 from an *A. pittii* isolate from Taiwan (99%) [13]. There were no *ISAba* sequences upstream of the *bla*_ADC-72_ gene. Multilocus sequence typing (MLST) of strain XM1570 revealed sequence type ST-70.

### Plasmid Characterization

We attempted to use polymerase chain reaction (PCR) to identify the replicon type of the plasmid harboring the *bla*_NDM-1_ gene [14], [15]. However, our attempts were unsuccessful. We made comparisons with the replicon sequences of *Enterobacteriaceae* [16], but could not confirm the replicon type of the plasmid sequence harboring the *bla*_NDM-1_ gene (AMXH01000087.1). The plasmid lacked known replicon genes indicating that it may has an unknown replication mechanism. The search of the 22 replicase genes in the whole genome sequence indicated that replicase gene *repB* was encoded by the other (90 kb) plasmid of strain XM1570 (CM001803.1), which had 100% nucleotide identity to p3ABAYE0002 (*A. baumannii*; NC010404.1). Furthermore, the *aci9* gene was found in the bacterial genome sequence, showing ∼87% nucleotide identity to that of the plasmid pA21 (*A. baumannii*; GU979001.1). Sequence analysis showed that the plasmid (CM001803.1) contained no antibiotic resistance genes but did contain other genes encoding proteins such as alcohol dehydrogenase, alkaline phosphatase, DNA-binding helix-turn-helix protein, inner membrane protein, Sel1 repeat protein, transcriptional repressor, integrase, site-specific recombinase, and transposase.

**Figure 1:**
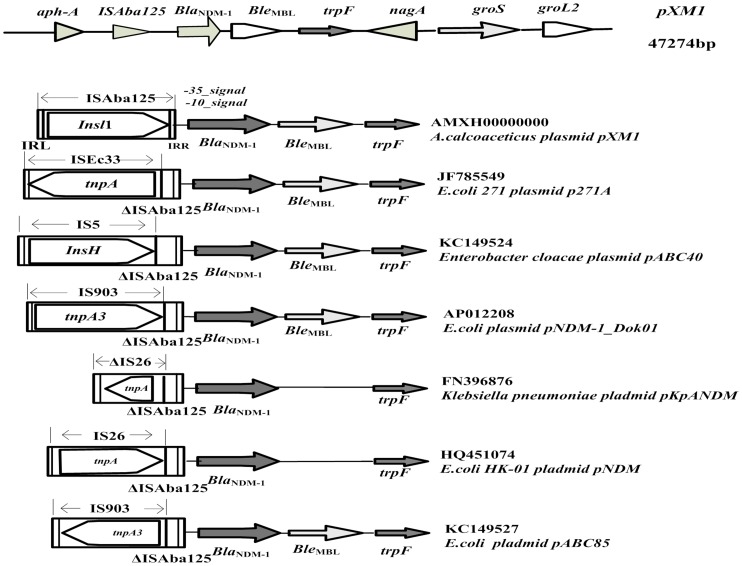
Schematic map representing the *bla*_NDM-1_-surrounding genetic sequences in the *A. calcoaceticus* XM1570. The *bla*_NDM-1_ genetic environment was compared with previously described structures of other NDM-1 plasmids (p271A, pABC40, pNDM-1_Dok01, pKpANDM, pNDM-HK, and pABC85). Different insertion elements were identified among these *Enterobacteriaceae.* Genes and their transcription orientations are represented by arrows. Δ indicates the truncated genes.

The *bla*_NDM-1_ genetic environment was systematically identified in our isolate, which was composed of the complete *ISAba125* fragment containing the *InsI1* transposase gene, the *bla*_NDM-1_ gene, the bleomycin resistance gene *ble*_MBL_, and the phosphoribosylanthranilate isomerase gene *trpF*. The *bla*_NDM-1_ gene exhibited 100% homology with that of the Hong Kong isolate HK-1 (*E. coli*) [17], and contained a 24-bp insertion sequence between the *bla*_NDM-1_and *trpF* genes compared with the India isolate KP05-506 (*K. pneumoniae*). We compared the genetic structures surrounding the *bla*_NDM-1_ of our isolate with that of isolates from India, Japan, and Hong-Kong (Figure 1), which showed that at least a remnant of the insertion sequence *ISAba125* was present upstream of the *bla*_NDM-1_ gene. The entire *ISAba125* element was only identified in our isolates, as the remnant *ISAba125* sequences of all others were disrupted by different mobile elements (*ISEc33*, *IS5*, *IS903* etc.), which contained the relevant transposase gene. Downstream of the *bla*_NDM-1_ gene, *ble*_MBL_ was systematically identified in most isolates, with the exceptions of FN396876 and HQ451074, and the *trpF* gene was identified in all isolates, which encode the isomerase that catalyzes the third step of synthesis of the amino acid tryptophan. As an isomerase, it can rearrange parts of the molecule without additions or deletions, indicating that the *trpF* gene was a vital component of the *bla*_NDM-1_ module.

### Conjugation and Transformation of *bla*_NDM-1_

The frequencies of transfer *in vitro* were ∼10^−3^ for *E. coli* DH5α:pBR322. Mass spectrometry (data not shown) identified the bacteria that grew on agar plates containing meropenem and tetracycline as *E. coli* DH5α. PCR demonstrated successful transfer of the *bla*_NDM-1_ gene from *A. calcoaceticus* to DH5α, and sequencing results confirmed the absence of genetic mutation within the gene. Plasmid isolation revealed that the transconjugant acquired only the *bla*_NDM-1_-containing plasmid (47.3kb size; AMXH01000087.1; data not shown). The *E. coli* F_25_ strain failed to grow under these conditions.

*A. calcoaceticus* (donor) and DH5α:pBR322 or F_25_:pBR322 (recipients) were cocultivated *in vivo* in mice. The discharged bacteria curves for *A. calcoaceticus*, DH5α, and the transconjugants showed that the number of discharged bacteria peaked at 2 h post-transfer. The frequencies of transfer *in vivo* for DH5α: pBR322 were ∼10^−6^. Mass spectrometry, PCR and sequencing identified the bacteria as *E. coli* DH5α harbouring the *bla*_NDM-1_-bearing plasmid. Consistent with our *in vitro* results, there was no evidence of *in vivo* transformation of the F_25_ recipient strain. The minimum bacteriostatic concentration of meropenem was 0.0625 µg/ml for the DH5α:pBR322 recipient and 256 µg/ml for DH5α:pBR322 successfully transferred *in vitro* and *in vivo*. The *E. coli* DH5α:pBR322 strain after successful transformation acquired drug resistance to meropenem, imipenem, ertapenem, ceftazidime, cefotaxime, and cefepime. Resistance to antibiotics other than β-lactams was not transferred to the transconjugant.

### Plasmid Elimination Analysis

The three methods used to eliminate bacterial plasmids were natural reproduction, sodium dodecyl sulfate (SDS) treatment, and nalidixic acid treatment. The plasmid-elimination rates were 3.32% (10/301) for natural reproduction, 83.78% (278/332) for SDS treatment, and 84.17% (298/354) for nalidixic acid treatment. The absence of the *bla*_NDM-1_-containing plasmid in elimination strains was confirmed by PCR and plasmid isolation. All plasmid-cured strains did not lose the other 93.8 kb plasmid (CM001803.1; data not shown). Antibiotic sensitivity was dramatically increased in elimination strains, which all exhibited a minimum bacteriostatic concentration for meropenem of 0.125 µg/ml, compared to 256 µg/ml for the clinical strain. The plasmid-cured strains lost resistance to carbapenem and some other β-lactams, including ceftazidime, cefotaxime, and cefepime, but maintained resistance to cefazolin, aztreonam, and ampicillin.

## Discussion

Currently, the majority of NDM-producing *Acinetobacter* spp. have been isolated in China and the Middle East [18], [19]. Most of the isolates were identified as *A. baumannii*. Other NDM-producing *Acinetobacter* spp. have been identified in Chinese isolates, including *A. pittii*, *A. lwoffii*, and *A. junii* [19]–[21]. In the present study, NDM-1-production in *A. calcoaceticus* was identified for the first time.

The inappropriate use of β-lactamase antibiotics is causing an increasing public health threat of multidrug-resistant bacteria and poses a considerable challenge to the treatment of clinical infections. The development of appropriate methods to eradicate drug-resistant bacteria has become a focus of recent research. Performance of 3 different plasmid-elimination tests to remove the antibiotic resistance of *A. calcoaceticus* showed that the methods with SDS and nalidixic acid treatment were more efficient than the natural reproduction elimination assay. The plasmid-eliminated strains had completely lost resistance to carbapenem and some other β-lactams, including ceftazidime, cefotaxime, and cefepime.

The presence of carbapenem hydrolyzing class D β-lactamases is the most important factor mediating carbapenem resistance in *Acinetobacter* spp. The carbapenemase genes *bla*_OXA-58_ and *bla*_OXA-23_ are distributed worldwide. The new intrinsic *A. calcoaceticus* enzyme OXA-417 gene was identified in the genome of our isolate. The coding sequence of the *bla*_OXA-417_ gene was prematurely terminated by insertion of *ISAba22*, which resulted in inactivation of the protein carbapenem-hydrolyzing oxacillinase [22]. Intrinsic *bla*_OXA_ genes, e.g. *bla*_OXA-51_ in *A. baumannii* only cause carbapenem resistance if an insertion sequence is present upstream (*ISAba1*-*bla*_OXA-51_). Given that no other insertion sequences are located upstream the gene, it is not clear if it can mediate carbapenem resistance even when functional.

Conjugation experiments by co-cultivation of the *A. calcoaceticus* strain with *E. coli* DH5α:pBR322 *in vitro* and *in vivo* in mice showed that the plasmid carrying the *bla*_NDM-1_ gene was successfully transferred to the recipient DH5α:pBR322 *in vitro* and *in vivo*. The minimum bacteriostatic concentration of meropenem for transduced bacteria was greatly increased, but was less than that of strain XM1570, suggesting that the degree of resistance was also dependent on physiological metabolic properties of the bacteria or by the contribution of additional genes to beta-lactam resistance, e.g. *bla*_ADC_ [23].

NDM-1 can efficiently hydrolyze a broad range of β-lactams, but not monobactams, such as aztreonam [18]. In our study, after successful transfer of the *bla*_NDM-1_-carrying plasmid, *E. coli* strain DH5α:pBR322 remained susceptible to aztreonam. The plasmid-cured strain maintained resistance to aztreonam. An increasing number of reports have focused on *Acinetobacter*-derived cephalosporinases (ADCs), which are common among *Acinetobacter* spp. [24]. Some ADC β-lactamases had lower MICs of cefotaxime and aztreonam as well. Aztreonam is a weak inducer and weak substrate, but it can be hydrolyzed if sufficient amounts of ADC β-lactamases are produced [25]. Some ADC β-lactamases (ADC-33) can hydrolyze high levels of ceftazidime, cefepime, and aztreonam, which encouraged the classification of this enzyme as an extended-spectrum AmpC (ESAC) [23]. Because of the insertional inactivation of *bla*_OXA-417_, we speculated that *bla*_ADC-72_ conferred resistance to aztreonam, cefazolin, and ampicillin in the plasmid-cured strain.

This is the first report of NDM-1 -positive *A. calcoaceticus* from China. Our study provides molecular evidence for the emergence and dissemination of multidrug-resistant bacteria. *bla*_NDM-1_ in *A. calcoaceticus* XM1570 located on a highly mobile plasmid of indeterminable replicon type, further underlining the wide spread of that resistance trait. The results of this study also suggest a potential treatment strategy that may help in controlling NDM-1 bacterial infection and transmission.

## Materials and Methods

### Case Report

Sputum was collected from a patient diagnosed with pneumonia treated at a hospital in Xiamen, Fujian Province, China, in 2010. The male patient was initially diagnosed at a community clinic with a cold, and treatment with injection of clarithromycin, imipenem, and cilastatin sodium had no effect. He was subsequently transferred to a hospital, where lung cancer with right pleural metastasis and pneumonia was confirmed. According to the drug-resistance pattern, the patient was treated with levofloxacin via intravenous drip. The symptoms caused by pneumonia were attenuated after 1 week of treatment.

### Bacterial Strains

A sputum culture yielded an *Acinetobacter* strain resistant to β-lactam antibiotics and carbapenems. The multidrug-resistant pathogen XM1570 was analyzed using the Vitek 32 microbial identification/susceptibility system (bioMérieux Inc., Durnam, NC, USA) with the GNI+ panel and matrix-assisted laser desorption ionization time-of-flight mass spectrometry.

### Antibiotic Susceptibility Testing

Susceptibilities of the *A. calcoaceticus* XM1570, transconjugants (DH5α:pBR322 strain after successful transformation), transformants, and plasmid-cured strains were tested using the BD automated microbiology system (Panel NMIC/ID-4) (see Table 1). Susceptibility of these bacterial strains to imipenem, meropenem, and ertapenem was assessed using the broth microdilution susceptibility method as defined by the Clinical and Laboratory Standards Institute (CLSI) document no. M100-S22, 2012 [26].

### Verification of *bla*_NDM–1_

A set of primer pairs was designed based on the open reading frame sequence of *bla*_NDM–1_ (product length, 831 bp): 5′-ATG GAA TTG CCC AAT ATT ATG CA-3′ (NDM-F) and 5′-TCA GCG CAG CTT GTC GGC CAT-3′ (NDM-R). The primers were synthesized by Beijing Genomics Institution. PCR-amplification was carried out in a total reaction volume of 50 µl containing 50 pmol of each primer and 2 µl of template (TaKaRa Bio, Inc., Osaka, Japan), as recommended by the manufacturer. The following specific cycling parameters were used: initial denaturation at 94°C for 3 min, followed by 35 cycles of denaturation at 94°C for 30 s, annealing at 53°C for 30 s, and extension at 72°C for 1 min, and a final extension step at 72°C for 7 min.

### Whole Genome Sequencing, Assembly and Analysis

Bacterial cells were collected by centrifugation at 6,000×*g* for 15 min and genomic DNA was extracted using a Wizard Genomic DNA purification kit (Promega, Madison, WI, USA) according to the manufacturer's instructions. Complete genomic sequencing was carried out at the Beijing Institute of Genomics, Chinese Academy of Sciences, using a HiSeq 2000 system (Illumina, Inc., San Diego, CA, USA). Ultimately, 410 and 210 Mb of high-quality data was generated from two libraries. In both cases, read lengths were 90 bp. The paired-end reads were first assembled *de novo* using a Short Oligonucleotide Analysis Package (SOAP denovo v1.05). *A. calcoaceticus* strain XM1570 contigs were next manually connected by reference to paired-end relationships at the 2-kb level. Prior to functional annotation, putative protein-coding sequences were identified using Glimmer 3.0 online (www.ncbi.nlm.nih.gov/genomes/MICROBES/glimmer_3.cgi) and homologies to sequences in publically available databases were identified using the Basic Local Alignment Search Tool (http://blast.ncbi.nlm.nih.gov/Blast.cgi). Gaps in plasmid sequences were bridged by PCR and confirmed by capillary sequencing. Sequences were submitted to Genbank (AMXH01000000). The MLST scheme was based on the allelic variations in seven housekeeping genes (*cpn60*, *fusA*, *gltA*, *pyrG*, *recA*, *rplB*, and *rpoB*) of the *Acinetobacter* whole genome sequences [27]. Allele sequences and sequence types (STs) were identified at http://www.pasteur.fr/recherche/genopole/PF8/mlst/Abaumannii.html.

### Conjugation and Transformation of *bla*_NDM-1_

To test the transferability of the *bla*_NDM-1_ gene, two recombinant bacteria (*E. coli* DH5α:pBR322 and *E. coli* F25:pBR322) were constructed and the conjugation-transfer assay was performed with *A. calcoaceticus* containing *bla*_NDM-1_ as the donor and *E. coli* DH5α or *E. coli* F_25_ as the recipient. For the *in vitro* conjugation and transformation assay, the donor and recipient bacteria were mixed at a ratio of 1:2 and then filtered through a microfiltration membrane, which was cultured in solid medium overnight with the cover containing the bacteria facing upward. Transconjugants were selected on solid lysogeny broth (LB) medium containing meropenem (16 µg/ml) and tetracycline (15 µg/ml), and verified by mass spectrometry (bacterial species) and PCR (*bla*_NDM-1_).

*In vivo* conjugation and transformation assays were performed in mice. The mice were denied food for 12 h and then fed donor and recipient bacteria mixed at a ratio of 1∶2. Gastric acid was neutralized with sodium hydrogen carbonate. Afterwards, the mice had access to food *ad libitum*. Mouse droppings were collected at 1, 2, 3, 4, and 5 h for analysis of excreted bacteria. The droppings were suspended in physiological saline. Transconjugants were selected by culturing suspensions on solid LB medium containing meropenem (16 µg/ml) and tetracycline (15 µg/ml) at suitable dilutions. Plasmid transfer was verified by mass spectrometry (bacterial species), PCR (*bla*_NDM-1_), and nucleotide sequencing. Antimicrobial resistance of the donor, recipient, and transconjugant strains was tested using the Phoenix100 system (BD Biosciences, San Jose, CA, USA) with the CLSI Broth Microdilution Susceptibility Method.

### Plasmid Elimination Study

Elimination experiments were carried out in broth culture. Plasmid curing was performed as described previously [28] with slight modifications. For the natural reproduction method, 50 µl of *A. calcoaceticus* was inoculated into 5 ml of LB medium and incubated at 37°C for 8 h. Then, 50 µl of bacterial suspension was inoculated into 5 ml fresh LB medium at 37°C and incubated for 8 h. This latter procedure was repeated five times. After serial dilution, the bacterial suspension was inoculated onto solid LB medium at 37°C and incubated for 12 h. All colonies were inoculated onto solid LB medium with or without meropenem, and incubated at 37°C for 12 h. Plasmid elimination was calculated as the difference in the amount of growth on the two media divided by the amount of growth on solid LB medium.

For the SDS treatment method, 50 µl of *A. calcoaceticus* suspension was inoculated into five replicate samples of 5 ml of LB medium, to which 200, 100, 50, 25 or 12.5 µl of 10% SDS was added and then incubated at 37°C for 8 h. An SDS concentration one-half of that in which growth was first observed was chosen as the working concentration for the elimination test. The following steps were then performed: (1) 50 µl of *A. calcoaceticus* was inoculated into 5 ml of LB medium containing SDS and incubated at 37°C for 12 h; (2) 50 µl of bacterial suspension was inoculated into 5 ml of fresh LB medium and incubated at 43°C for 8 h; (3) repeat step 1; (4) repeat step 2; and (5) repeat step 1.

For the nalidixic acid treatment method, 50 µl of *A. calcoaceticus* suspension was inoculated into five replicate samples of 5 ml of LB medium, to which 10, 5, 2.5, 1.25 or 0.625 µl of 50 mg/ml nalidixic acid was added and the samples were incubated at 37°C for 12 h. A nalidixic acid concentration one-half of that in which growth was first observed was chosen as the working concentration for the elimination test. Then, 50 µl of *A. calcoaceticus* suspension was inoculated into 5 ml of LB medium and incubated at 37°C for 8 h. This procedure was repeated five times. Plasmid elimination was calculated as for natural reproduction. Plasmids of *A. calcoaceticus* XM1570 and the plasmid-cured derivative strain were isolated using the Qiagen Large-Construct Kit (Qiagen, Hilden, Germany). The absence of plasmids in elimination strains was confirmed by PCR. Antibiotic-resistance profiles of plasmid-harboring and plasmid-cured strains were tested using the BD Phoenix100 system. The MICs of carbapenems were confirmed by the CLSI Broth Microdilution Susceptibility Method [26].

### Ethics Statement

The study protocol was approved by the Review Board of the Academy of Military Medical Sciences and Beijing Institute of Genomics. The patient provided oral informed consent because he was unable to write. The oral consent was documented by the attending physician. This consent procedure was approved by the Review Board of the Academy of Military Medical Sciences and Beijing Institute of Genomics. All animal experiments were conducted in accordance with the accepted standards of the Animal Care and Use Committee of Academy of Military Medical Sciences, China. The animal study protocol was approved by the Animal Care and Use Committee of Academy of Military Medical Sciences, China. All efforts were made to minimize suffering.
